# Efficacy and safety of Lian-Ju-Gan-Mao capsules for treating the common cold with wind-heat syndrome: study protocol for a randomized controlled trial

**DOI:** 10.1186/s13063-016-1747-9

**Published:** 2017-01-05

**Authors:** Shengjun Wang, Hongli Jiang, Qin Yu, Bin She, Bing Mao

**Affiliations:** 1 Department of Integrated Traditional Chinese and Western Medicine, West China Hospital of Sichuan University, Chengdu, 610041 Sichuan Province China; 2 National Clinical Trials Agency/GCP Center, West China Hospital of Sichuan University, Chengdu, 610041 Sichuan Province China

**Keywords:** Lian-Ju-Gan-Mao capsules, Common cold, Wind-heat syndrome, Randomized controlled trial, Chinese herbal medicine

## Abstract

**Background:**

The common cold is a common and frequent respiratory disease mainly caused by viral infection of the upper respiratory tract. Chinese herbal medicine has been increasingly prescribed to treat the common cold; however, there is a lack of evidence to support the wide utility of this regimen. This protocol describes an ongoing phase II randomized controlled clinical trial, based on the theory of traditional Chinese medicine (TCM), with the objective of evaluating the efficacy and safety of Lian-Ju-Gan-Mao capsules (LJGMC), a Chinese patent medicine, compared with placebo in patients suffering from the common cold with wind-heat syndrome (CCWHS).

**Methods/design:**

This is a multicenter, randomized, double-blind, placebo-controlled phase II clinical trial. A total of 240 patients will be recruited and randomly assigned to a high-dose group, medium-dose group, low-dose group, and placebo-matched group in a 1:1:1:1 ratio. The treatment course is 3 consecutive days, with a 5-day follow-up. The primary outcome is time to all symptoms’ clearance. Secondary outcomes include time to the disappearance of primary symptoms and each secondary symptom, time to fever relief, time to fever clearance, and change in TCM symptom and sign scores.

**Discussion:**

This trial is a well-designed study according to principles and regulations issued by the China Food and Drug Administration (CFDA). The results will provide high-quality evidence on the efficacy and safety of LJGMC in treating CCWHS and help to optimize the dose for the next phase III clinical trial. Moreover, the protocol presents a detailed and practical methodology for future clinical trials of drugs developed based on TCM.

**Trial registration:**

Chinese Clinical Trial Registry, ChiCTR-IPR-15006504. Registered on 4 June 2015.

**Electronic supplementary material:**

The online version of this article (doi:10.1186/s13063-016-1747-9) contains supplementary material, which is available to authorized users.

## Background

The common cold, a conventional term for a mild upper respiratory tract infection (URTI), is a common and frequent respiratory disease mainly caused by a virus [1, 2]. Adults may have about two to four episodes annually, whereas children may experience up to six to eight colds per year [2, 3]. Although the common cold is usually mild and self-limiting, with a mean duration of 7–10 days [3, 4], its economic burden on society is significant in terms of visits to health care providers, treatments, and absences from work and school [5, 6]. Besides, in some cases, viral pathogens may spread to adjacent organs, resulting in different clinical manifestations, and, occasionally, colds predispose to bacterial complications [7].

Symptoms of a cold often include fever, headache, fatigue, nasal stuffiness and discharge, sneezing, sore throat, and cough [4]. Although great progression has been achieved in Western medicine, there are still no clinically proven “gold standard” medications directly targeting the causative pathogen for treating a cold. Current treatments are limited to symptomatic supportive options that maximize the comfort of patients by reducing symptom severity and limiting the occurrence of complications; these treatments include antihistamines, decongestants, expectorants, antitussives, antipyretics, and analgesics. However, a series of adverse reactions to these drugs, such as drowsiness, dizziness, dry mouth, digestive system dysfunction, and epistaxis, occasionally occur [8–10]. Accordingly, Chinese herbal medicine as well as zinc and/or vitamin C, garlic, echinacea and vitamin D_3_, as complementary and alternative therapies, have been developed and used in patients with the common cold [11–15].

Traditional Chinese medicine (TCM) is a unique, well-established system of medicine with a history of several thousand years, which has been proven effective in the treatment of many diseases, especially the common cold. As the most important component of TCM, Chinese herbal medicine mainly derives from plants and usually incorporates one or more herbs to treat diseases. The common cold can be divided into wind-cold, wind-heat, or summer-heat and dampness syndromes according to its TCM symptoms and signs, such as fever, aversion to cold, tongue proper, tongue coating, and condition of the pulse [16], which is called syndrome differentiation (*bian zheng lun zhi*). Common cold with wind-heat syndrome (CCWHS) is the most common type and is primarily characterized by fever, mild aversion to cold, sore throat, red tongue, thin yellow tongue coating, and a floating and fast pulse.

Lian-Ju-Gan-Mao capsules (LJGMC) are a novel Chinese patent medicine, manufactured by Tasly Pharmaceutical Co., Ltd. (Tianjin, China), composed of *Yangerju (Inula cappa)*, *Chuanxinlian (herba andrographis)*, and *Shengshigao (Gypsum)* (Table 1). The drug prescription has been used as a decoction in clinical practice for years in Southwest Guizhou Autonomous Prefecture Hospital of TCM, and it has demonstrated notable efficacy in lowering body temperature, improving patients’ symptoms, and shortening the duration of the cold, with few side effects. Preclinical pharmacological and toxicological studies on animals showed that LJGMC was effective in clearing heat, relieving pain, reducing inflammation, and alleviating cough, as well as producing antiallergic, antibacterial, and antiviral effects with no evidence of toxic effects [17–20].

**Table 1:** Pharmacological effects of each ingredient in a Lian-Ju-Gan-Mao capsule

Ingredient	Latin name	Pharmacological effects based on TCM
Yangerju	Inula cappa	Dispels wind heat, removes toxicity, and soothes throat
Chuanxinlian	Herba andrographis	Clears heat and toxicity
Shengshigao	Gypsum	Clears heat and purges lungs

*TCM* Traditional Chinese medicine

This phase II clinical trial is well designed to evaluate the efficacy and safety of LJGMC compared with placebo in patients with CCWHS. Additionally, it is expected to find a dose-efficacy relationship and to help determine the optimum dose for the next phase III clinical trial. Moreover, the protocol presents a detailed and practical methodology for future clinical trials of patent drugs developed based on TCM.

## Methods/design

### Study design

**Fig. 1:**
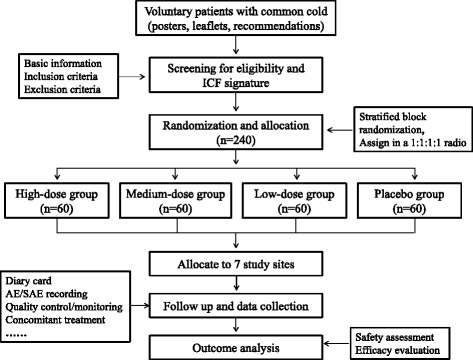
Study flow chart. *AE* adverse event, *SAE* serious adverse event

This study is a multicenter, parallel group, double-blind, prospective, randomized, placebo-controlled phase II clinical trial. A total of 240 patients will be recruited and randomly allocated into treatment groups or placebo group. All patients will receive the treatment for 3 consecutive days, with a 5-day follow-up. Efficacy and safety data will be collected throughout the whole study. The flow chart is shown in Fig. 1.

### Ethics

This trial has been authorized by the China Food and Drug Administration (CFDA) (Approval Number 2012 L01822) and registered in the Chinese Clinical Trial Registry (ChiCTR-IPR-15006504). In addition, the study will be conducted in compliance with the Declaration of Helsinki, Good Clinical Practice (GCP) guidelines, and the laws and regulations of clinical trials issued by the CFDA. The protocol, informed consent form, and recruitment poster were reviewed and approved by the West China Hospital of Sichuan University Clinical Trials and Biomedical Ethics Committee (number TCM-2015-03) and the ethics committees of the other six hospitals. The investigators in all seven trial centers are qualified and well trained. All eligible participants will be fully informed about the protocol and will sign an informed consent form prior to participation. The privacy and data of all participants will be protected, and their safety will be greatly guaranteed.

### Participants and recruitment

We will recruit eligible participants by advertising on hospital notice boards, sending leaflets, or being recommended by doctors in outpatient clinics. A total of 240 patients will be recruited at the following seven hospitals: (1) West China Hospital of Sichuan University, (2) Shuguang Hospital Affiliated to Shanghai University of TCM, (3) First Affiliated Hospital of Guiyang College of TCM, 4) First Affiliated Hospital of Tianjin University of TCM, (5) Second Affiliated Hospital of Tianjin University of TCM, (6) Ruikang Hospital Affiliated to Guangxi University of TCM, and (7) First Affiliated Hospital of Guangzhou University of TCM. Forty patients will be recruited at each of the first two centers and 32 at each of the latter ones.

#### Inclusion criteria

For inclusion, participants should fulfill all the following criteria:

1. Diagnosis of common cold according to Western medicine
2. Diagnosis of common cold with wind-heat syndrome according to TCM
3. Aged between 18 and 65
4. Presenting within 36 hours after symptoms onset
5. Willingness to participate and to sign the informed consent form.

#### Exclusion criteria

Participants with any of the following conditions will be excluded:

1. Patients with acute herpetic pharyngitis, acute viral pharyngitis, acute herpetic laryngitis, acute viral laryngitis, acute conjunctivitis or acute tonsillitis, pneumonia, and other diseases
2. Patients with severe primary diseases of cardiovascular, lung, kidney, or hematopoietic system or abnormal electrocardiogram with clinical significance
3. Patients with liver function (such as alanine aminotransferase [ALT] and aspartate aminotransferase [AST]) 1.5 times higher than the normal upper limit, abnormal serum creatinine, positive urine protein qualitative test, white blood cell count <3.0 × 10^9^/L or >10.0 × 10^9^/L, and/or neutrophil percentage >80%
4. Patients who had used other drugs to treat common cold after the onset of the disease
5. Temperature ≥39.0 °C
6. Women who are pregnant or preparing to become pregnant or breast feeding women
7. Allergic condition or allergy to the drug composition(s)
8. Patients with mental or legal disabilities
9. Patients who participated in clinical trials of other drugs within the past 3 months
10. Patients who are not suitable for the trial as decided by the researchers.

#### Withdrawal criteria

The withdrawal criteria include the following:

1. Worsening conditions during the trial, including no body temperature decline, a rising body temperature higher than 39.0 °C, or worsening symptoms in 48 hours
2. Experiencing anaphylaxis or serious adverse events during the trial
3. The study drug is not taken as required, or is taken at doses <80% or >120% of the requirement
4. Quitting the clinical trial voluntarily.

#### Early suspension or termination of the entire study

The trial will be suspended early or terminated for the following reasons:

1. A serious adverse event (SAE) which is probably or definitely related to the test drug occurs or more than half of the population in any of the groups experience adverse drug reactions.
2. The efficacy of the test drug is found to be poor or even ineffective.
3. The protocol is flawed, or there is significant deviation from the protocol.
4. The pharmaceutical supervisory and administrative department decides to terminate the study reasonably.
5. The sponsor decides to terminate the trial due to management or financial problems.

### Diagnostic criteria

A diagnosis of the common cold in Western medicine is established according to *Practice of Internal Medicine* (2013, 14^th^ edition) [21]. A TCM diagnosis of CCWHS is based on the chapter common cold (wind-heat syndrome) of *Internal Medicine of TCM* [22] and *TCM syndrome and sign diagnostic criteria for common cold* [23]. The TCM diagnostic criteria for CCWHS are listed in Table 2. To be diagnosed with CCWHS, patients should have all the primary symptoms and at least three of the secondary symptoms, as well as TCM signs for the tongue and pulse.

**Table 2:** Diagnostic criteria of common cold based on TCM

Category	Symptoms and signs
Primary symptoms	Fever, mild aversion to cold, sore throat
Secondary symptoms	Weak limbs, nasal congestion, running nose, thirst, cough
Signs for the tongue	Red tongue with thin yellow coating
Signs for the pulse	Floating and rapid pulse

*TCM* Traditional Chinese medicine

### Randomization and blinding

A total of 240 eligible participants will be randomly allocated to the high-dose group, medium-dose group, low-dose group, or placebo group in a 1:1:1:1 ratio. Randomized sequences of each center and every packed drug were generated by an independent professor at the Drug Clinical Research Center of Shanghai University of TCM, using a stratified block randomization method with 30 blocks of block size 8 based on the PROC PLAN function of the SAS 9.2 software analysis system (SAS Institute, Cary, NC, USA).

Randomization sequences will be concealed in lightproof, sealed envelopes kept by a specified project manager and the sponsor, who are not involved in the recruitment, intervention, assessment, or statistical analysis. The treatment allocation will be blinded to the participants and investigators throughout the study, and the outcome assessors and statisticians will not be involved in the participants’ screening and allocation. Each patient will receive a unique randomized number corresponding to the drug according to the group allocation. Furthermore, the intervention group type will be replaced by the letter A, B, C, or D as blind codes of allocation. The blind codes will not be disclosed until the statistical analysis is completed. An emergency envelope has been prepared for each randomized number in each center and is to be opened for treatment only in case of medical emergency which is most probably caused by the study drug.

### Interventions

LJGMC and the placebo are provided and manufactured by Tasly Pharmaceutical Co., Ltd. (Tianjin, China). Each LJGMC or placebo weighs 0.28 g. The production batch number is 20150101. All the drugs are packaged uniformly with the same labels. Each package, which contains 3 plus 1 days’ dosage, must be sealed and kept in a cool, dry place. The placebo, which contains no effective ingredients to treat the common cold, is almost identical to the study drug in appearance, smell, and taste. A clearly visible label on each package states “FOR TRIAL USE ONLY” and other information: name, dose, dosing schedule, indication of storage condition, expiration date, and the manufacturer’s name. An independent drug manager in each center is in charge of receiving, handling, storing, and dispensing the drugs.

Patients in the high-dose group, medium-dose group, low-dose group, or placebo-matched group will receive, respectively, 1.12 g LJGMC, 0.56 g LJGMC plus 0.56 g placebo, 0.28 g LJGMC plus 0.84 g placebo, or 1.12 g placebo each time, three times per day. All medications will be taken orally with 200 ml warm water. The treatment duration is 3 consecutive days, with a 5-day follow-up. During the study, patients will be visited four times by the investigators. Details of the study procedures are given in Table 3.

**Table 3:**
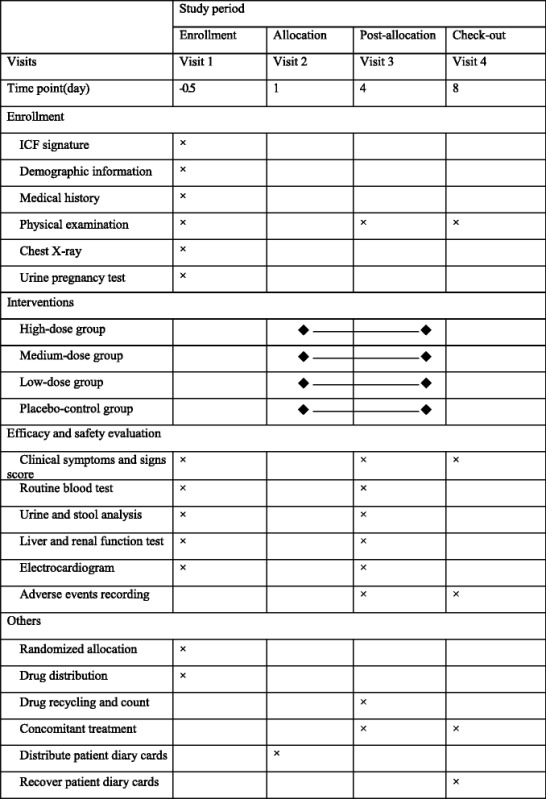
Study schedule for patients

*ICF* informed consent form

### Concomitant treatments and forbidden drugs

Patients will be allowed to continue using medications for underlying conditions, such as hypertension or diabetes. The name, dosage, and duration of any concomitant medication must be recorded carefully in the case report form (CRF). If the body temperature of a participant rises to higher than 39.0 °C for longer than 2 hours or becomes ≥39.5 °C, physical cooling and/or paracetamol can be used to kill the fever. During the study period, any other Western or Chinese therapy or medication that may affect the study results is prohibited. Once forbidden drugs are used, the patient will be withdrawn from the study.

### Safety evaluation

Adverse events (AEs) concerning clinical symptoms and signs as well as laboratory tests will be documented. Every AE will be recorded in detail, and prompt, proper treatment will be given by researchers and therapists at each center. If a serious adverse event (SAE) presents, a decision on whether the participant needs to be withdrawn from the study will be made by the principal investigator and ethics committee. The causality and relationship between AEs/SAEs and the study drug will also be analyzed and evaluated on the basis of standard operation procedures (SOPs) of the CFDA, which define causality and relationship as clearly related, probable, possible, doubtful, and clearly not. If an SAE occurs, the investigator must record it in detail on the SAE form, and it should be reported to the principal investigator, the CFDA, ethics committee, and the sponsor within 24 hours. A statistician will analyze the frequency and proportion of AEs/SAEs. Continuous care will be available until subjects who suffer harm from the participation recover, and proper compensation will also be given.

Primary vital signs, physical examinations, and some laboratory tests will be performed both before and after treatment for safety assessment. Primary vital signs include body temperature, blood pressure, heart rate, and respiration rate. Laboratory tests include routine blood, urine, and stool analysis, liver function test (ALT, AST, alkaline phosphatase (ALP), serum total bilirubin (STB), and gamma-glutamyl transpeptidase (γ-GT)), renal function test (serum creatinine, microalbuminuria, and serum cystatin C), and electrocardiogram. Also, before treatment, subjects who are suspected to have a lower respiratory tract infection need have a chest X-ray, and female patients with the potential to become pregnant must take a urine pregnancy test.

### Efficacy assessment

#### Primary outcome

The primary outcome is the time to all symptoms’ clearance, which is defined as the length from study enrollment to the time when the symptoms completely disappear. Each participant will be instructed to record any change in symptoms in their patient diary.

#### Secondary outcomes

Secondary outcomes include time to primary symptoms and each secondary symptom disappearance, time to fever relief, time to fever clearance, and change in TCM symptom and sign scores.

Time to fever relief is defined as the time from the first dosing to time when the body temperature drops by at least 0.5 °C. Time to fever clearance is defined as the time from the first dose administration to the time when the body temperature drops below 37.3 °C and lasts for 24 hours.

In this study, all patients will be required to record their body temperature in the patient diary every 2 hours within the first 12 hours and every 4 hours during the following 12 hours. After that, if the temperature is ≥37.3 °C, it should be recorded four times at a fixed time every day, and if the temperature is lower than 37.3 °C, then there is no need to record it.

### Change in TCM symptom and sign scores

The TCM symptom scoring system used in the study follows the *Guidelines for Clinical Research of New Chinese Medicine* [24], in which all symptoms are graded (Table 4). The change in cumulative TCM symptom and sign scores is assessed by the percentage of symptom and sign scores reduction (PSSSR), which is calculated according to the following formula: PSSSR = (scores before treatment – scores after treatment)/scores before treatment*100%. A value greater than 50% is defined as clinically effective.

**Table 4:** Symptom and sign scoring system

Symptom	Score			
Primary	None (0)	Mild (2)	Moderate (4)	Severe (6)
Fever	<37.3 °C	37.3–37.9 °C	38.0–38.4 °C	≥38.5 °C, <39.0 °C
Pharynx uncomfortable	–	Dry pharynx	Burning pharynx	Pharynx pain
Secondary	None (0)	Mild (1)	Moderate (2)	Severe (3)
Stuffy nose	–	Heavy sound	Sometimes	Sustained
Running nose	–	Occasionally	Sometimes	Sustained
Thirsty	–	No need for water	Sometimes need water	Need water frequently
Cough	–	Occasionally	Sometimes	Frequently
Headache	–	Occasionally, mild	Sustained, moderate	Heavy, can’t insist on work
Limb pain/soreness	–	Weak, uncomfortable	Painful	Terrible, can’t stretch freely
Cumulative symptom score	Primary symptom scores + secondary symptom scores			
Sign	None (0)	Mild (1)	Moderate (2)	Severe (3)
Pharyngeal hyperemia/swelling	–	Pharyngeal mucosa hyperemia	Pharyngeal mucosa, pharynx palatine arches and uvula hyperemia	Pharyngeal swelling and heavy secretion
Total score	All symptom scores + sign score			

– No symptom

### Sample size

The sample size calculation is based on a comparison between the intervention group and the placebo-controlled group. The mean duration of all symptoms for the common cold was estimated to be 7 ± 3 days [7, 25], and reduction in the duration of all symptoms by 2 days is assumed to be clinically significant. Using Power Analysis and Sample Size (PASS) 11.0 with 90% power (1–β) and α = 0.05 (two-sided), the sample size needs to be 48 cases for each group. Considering a drop-out of 20%, we decided to recruit 60 subjects in each group. According to the latest edition of Provisions for Drug Registration issued by the CFDA on phase II clinical trials, the total sample size was set to be 240 patients.

### Data management and statistical analysis

The Drug Clinical Research Center of Shanghai University of TCM is in charge of data statistical analysis. The data entry will be performed by two independent data administrators using the software EpiData 3.1 based on the CRFs, which should have been checked and reviewed for accuracy and consistency by the clinical research associate (CRA) and investigators. Finally, the database will be locked and analyzed under the agreement and confirmed review of the sponsor, principal investigator, and statistician.

The full analysis set (FAS), in which patients should be dosed with LJGMG or placebo at least once with clinical observation record in the study, is the primary analysis set according to the intention-to-treat (ITT) principle using the last observation carried forward (LOCF) approach for missing values. All subjects without any major protocol deviations will be included in the per-protocol set (PPS). An efficacy assessment will be carried out using the FAS and PPS. A safety evaluation will be conducted for subjects who have experienced at least one study visit and have safety data, which is defined as the safety set (SS).

All continuous and normally distributed data are presented as mean ± standard deviation, and median with range for non-normal data. Categorical data are summarized by frequency counts and percentages. Baseline balance among groups will be conducted by performing a chi-squared test or an analysis of variance (ANOVA). Time to all symptoms’ clearance, time to primary symptoms and each secondary symptom disappearance, time to fever relief, and time to fever clearance will be estimated and compared by the log-rank test. A Kaplan-Meier survival curve will be constructed, and the median time will be provided separately for each group with a two-sided 95% confidence interval. Comparisons among groups of TCM symptom and sign scores will be conducted using ANOVA and the Bonferroni method. All data will be processed by a professional statistician using SAS 9.2 software (SAS Institute, Cary, NC, USA); a two-sided *P* value of <0.05 is considered to be statistically significant.

### Quality control and monitoring

Each trial center has a project manager who is responsible for the quality of research. All investigators are qualified and well trained. The principal investigator in each center will be responsible for the trial conductance based on the SOP. During the whole course, independent trial inspectors will pay regular visits to each center, check source documents and CRFs, and supervise the research to make sure it complies with the protocol. Any shortcomings or problems found by the inspectors should be improved.

To ensure maximum compliance with drug taking and diary recording, a series of measures will be taken which include reminding subjects of their appointments, conducting attentive follow-up by phone or email, and making follow-up appointments at each participant’s convenience.

## Discussion

To date, no proven effective drugs or methods in clinical practice can cure the common cold. Chinese herbal medicine has been widely used to treat colds in China for a long time and has gradually been accepted and applied in many other countries. However, evidence-based efficacy and safety data on Chinese herbs are limited, because the studies have used small sample sizes or are of poor quality [26].

It is widely recognized that a randomized placebo-controlled trial is the gold standard to evaluate the safety and efficacy of a drug or treatment method. LJGMC, prescribed as a decoction, has been proven efficient and safe in clinical practice for years in Southwest Guizhou Autonomous Prefecture Hospital of TCM. To support its wide use, we have designed this multicenter, double-blind, placebo-controlled, randomized clinical trial in accordance with the Consolidated Standards of Reporting Trials (CONSORT) and Standard Protocol Items: Recommendations for Interventional Trials (SPIRIT) guidelines [27, 28] and the “One study, one primary outcome” methodology for clinical trials published by the CFDA. The SPIRIT checklist is given in Additional file 1. The study is expected to provide high-quality evidence in evaluating the efficacy and safety of LJGMC in treating CCWHS, and it may help to optimize the dose for the next phase III clinical trial based on the four parallel set groups. If proven beneficial, it provides further proof for people to use this inexpensive, easily accessible alternative choice to treat the common cold. Moreover, the protocol presents a detailed and practical methodology for future clinical trials of drugs developed based on TCM.

### Trial status

The study is currently in the process of recruiting participants in seven trial centers.

## Additional file

Additional file 1: SPIRIT 2013 checklist: recommended items to address in a clinical trial protocol and related documents. (DOCX 56 kb)

## Abbreviations

AE: Adverse event
ALP: Alkaline phosphatase
ALT: Alanine aminotransferase
ANOVA: Analysis of variance
AST: Aspartate aminotransferase
CCWHS: Common cold with wind-heat syndrome
CFDA: China Food and Drug Administration
CONSORT: Consolidated Standards of Reporting Trials
CRA: Clinical research associate
CRF: Case report form
FAS: Full analysis set
GCP: Good Clinical Practice
ITT: Intention-to-treat
LJGMC: Lian-Ju-Gan-Mao capsules
LOCF: Last observation carried forward
PASS: Power Analysis and Sample Size
PPS: Per-protocol set
PSSSR: Percentage of symptom and sign score reduction
SAE: Serious adverse event
SOP: Standard operation procedure
SPIRIT: Standard Protocol Items Recommendations for Interventional Trials
SS: Safety set
STB: Serum total bilirubin
TCM: Traditional Chinese medicine
URTI: Upper respiratory tract infection
γ-GT: Gamma-glutamyl transpeptidase
